# Nanomilling of Drugs for Bioavailability Enhancement: A Holistic Formulation-Process Perspective

**DOI:** 10.3390/pharmaceutics8020017

**Published:** 2016-05-20

**Authors:** Meng Li, Mohammad Azad, Rajesh Davé, Ecevit Bilgili

**Affiliations:** Otto H. York Department of Chemical, Biological and Pharmaceutical Engineering, New Jersey Institute of Technology, Newark, NJ 07102, USA; ml262@njit.edu (M.L.); mazad@mit.edu (M.A.); dave@njit.edu (R.D.)

**Keywords:** drug nanoparticles, wet stirred media milling, stabilization, process parameters, process modeling

## Abstract

Preparation of drug nanoparticles via wet media milling (nanomilling) is a very versatile drug delivery platform and is suitable for oral, injectable, inhalable, and buccal applications. Wet media milling followed by various drying processes has become a well-established and proven formulation approach especially for bioavailability enhancement of poorly water-soluble drugs. It has several advantages such as organic solvent-free processing, tunable and relatively high drug loading, and applicability to a multitude of poorly water-soluble drugs. Although the physical stability of the wet-milled suspensions (nanosuspensions) has attracted a lot of attention, fundamental understanding of the process has been lacking until recently. The objective of this review paper is to present fundamental insights from available published literature while summarizing the recent advances and highlighting the gap areas that have not received adequate attention. First, stabilization by conventionally used polymers/surfactants and novel stabilizers is reviewed. Then, a fundamental understanding of the process parameters, with a focus on wet stirred media milling, is revealed based on microhydrodynamic models. This review is expected to bring a holistic formulation-process perspective to the nanomilling process and pave the way for robust process development scale-up. Finally, challenges are indicated with a view to shedding light on future opportunities.

## 1. Introduction

The number of poorly water-soluble drug candidates coming out of drug discovery has increased tremendously over the past few decades [1,2,3,4]. Their formulation into efficacious dosage forms presents various challenges [2], and preparation of drug nanoparticles or nanocrystals is one way to formulate such drugs because size reduction of drug crystals increases the specific surface area, which can improve the dissolution rate of such drugs [5,6,7] according to the Noyes–Whitney equation [8], and, in turn, their bioavailability. Moreover, ultrafine particles especially those with sizes less than 100 nm tend to show higher saturation solubility, which also enhances the dissolution rate, and this phenomenon can be explained via the Kelvin and the Ostwald–Freundlich equation [9]. Hence, a nano-formulation approach to bioavailability enhancement largely relies on reduced crystal size with higher specific surface area and, to smaller extent, enhanced saturation solubility [10]. There are several approaches to producing drug nanoparticles such as wet media milling (referred to as nanomilling in the context of this review paper with a focus on nanosuspension preparation), homogenization, liquid antisolvent precipitation, melt emulsification, precipitation using supercritical fluid, evaporative precipitation, and micro-emulsions, and these approaches have been covered in several review papers [11,12,13,14,15,16]. Their advantages and disadvantages have also been discussed and compared [17,18,19,20].

**Figure 1:**
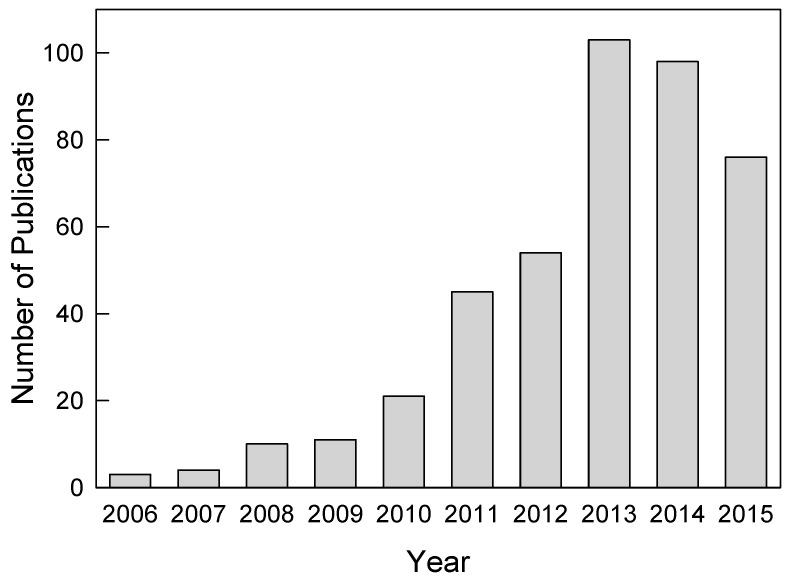
The number of published scientific papers in the period from 2006 to 2015 which reported the use of wet milling for poorly soluble drugs (source: Scopus database, key words: “poorly soluble drug” or “BCS Class II” or “insoluble drug” or “slightly soluble drug” or “drug nanoparticle” or “drug nanocrystal” and “wet milling”).

Milling is one of the most common pharmaceutical unit operations, which reduces drug particle size and increases the surface area. Nanomilling refers to the reduction of the drug particle size below 1000 nm by wet media milling [21,22,23], and the intermediate product is a drug nanoparticle suspension, conveniently referred to as a nanosuspension throughout this paper. Wet media milling is an organic solvent-free process and has several distinct advantages such as tunable and relatively high drug concentration, low excipient side effects, ability to run continuously, *etc.*, and can be universally applied to most drug candidates with poor water-solubility [24]. Nanosuspensions also offer the advantage of higher mass packing (and thus higher dose) per injection volume and improved physical stability via use of stabilizers such as polymers and/or surfactants [25,26]. Hence, the production of nanosuspensions via wet media milling has proved to be an effective method to overcome bioavailability challenges of several poorly water-soluble drugs. With all the above mentioned benefits, nanosuspensions can be used for various oral, injectable, and nebulized inhalation delivery applications [27], yet they have been mostly dried into powders in the production of standard solid dosage forms such as capsules, tablets, sachets (e.g., References [28,29,30,31,32,33,34,35]), and, recently, polymeric strip films [36,37,38,39,40]. For all the aforementioned reasons, the interest in wet media milling of poorly water-soluble drugs has increased over the years. Figure 1 presents the number of scientific publications in the last decade and shows the growing interest with some saturation in the last few years.

**Figure 2:**
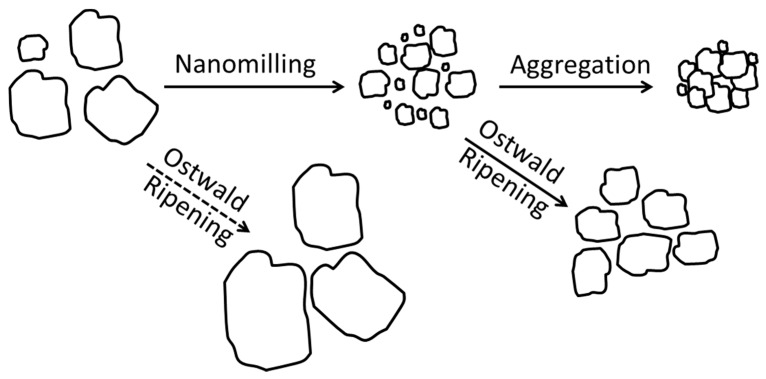
Schematic of possible mechanisms operating during the wet media milling of drugs.

A schematic of possible mechanisms occurring during the wet media milling of drugs is shown in Figure 2. Particle size during milling generally depends on (i) process-equipment parameters; (ii) mechanical and physico-chemical properties of drug particles; and (iii) physical stability of the milled suspension, *i.e.*, mitigation of aggregation and/or Ostwald ripening in the presence of various stabilizers [41,42]. Preparation of a drug nanosuspension with desired particle size and adequate storage stability entails selecting a proper stabilizer formulation and effective process-equipment parameters for the wet media milling process. The selection of optimal stabilizer formulation is a laborious and resource-demanding task, yet an important one with potentially serious consequences. A poorly formulated drug nanosuspension may undergo aggregation, Ostwald ripening, fast sedimentation of particles, and cake formation during milling/storage, which will lead to various issues in downstream processing of the respective suspensions and poor product performance from the final dosages such as unexpectedly slow dissolution [28,30,33,36,37,38,40]. Obviously, potential particle size increase/growth during milling and storage can lead to loss of high surface area associated with the drug nanoparticles, which reduces the significant benefits intended from the nanomilling process.

Several nanosuspension-based formulations have been either in development or in the market for more than two decades [43,44]. The marketed products that make use of wet media milling include Rapamune^®^ (Pfizer (Wyeth), New York City, NY, USA), Emend^®^ (Merck, Kenilworth, NJ, USA), Tricor^®^ Lipanthyl^®^ (Abbott Laboratories, Fournier Pharma, Montréal, QC, Canada), Megace^®^ ES (PAR Pharmaceuticals, Woodcliff Lake, NJ, USA), and Invega^®^ Sustenna^®^ Xeplion^®^ (Janssen, Beerse, Belgium). Interestingly, up to now, only a few academic publications [45,46,47] have investigated the impact of process parameters on the production of drug nanoparticles systematically and fundamentally with some models. Process parameters such as stirrer speed, bead loading, drug concentration, and bead size can significantly affect the breakage rate and cycle time required in a wet media milling process.

**Figure 3:**
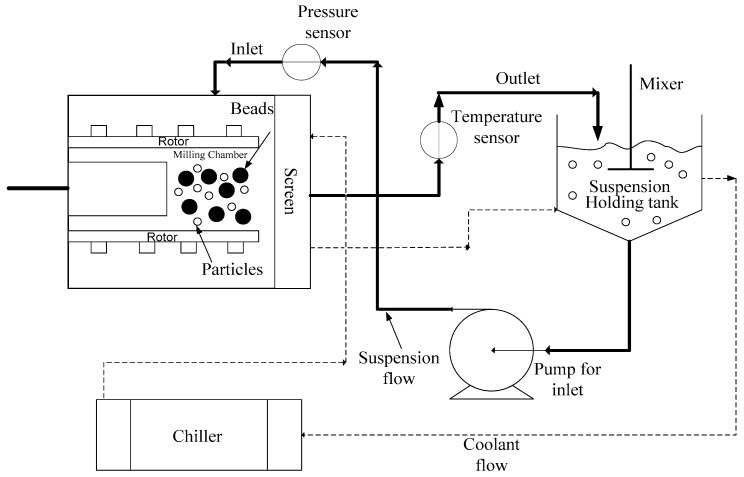
Schematic of a wet stirred media mill with recirculation mode of operation [45] (reprinted with permission. Copyright Elsevier 2014).

Among the wet media milling processes which use various equipment such as stirred mills, planetary mills, ball mills, *etc.*, the wet stirred media milling (WSMM) has been the most widely used and industrially most relevant process [47,48]. Hence, unless otherwise indicated in this review paper, wet media milling to prepare drug nanoparticles, also referred to as nanomilling, was considered mostly in the context of WSMM. A schematic of the WSMM process in recirculation mode is shown in Figure 3.

In the recirculation mode of WSMM operation, the suspension circulates from the holding tank passing through the milling chamber, exiting through the screen, and returning to the holding tank, while the milling media (beads) are retained inside the milling chamber by the screen. High speed rotation of the rotor/stirrer induces turbulent motion in the suspension, and turbulent energy dissipates during frequent bead–bead collisions [49]. The particles are subjected to stress, which is concentrated on the cracks already present in the material and causes crack propagation leading to fracture [50].

Unfortunately, the majority of the review papers published so far focused mostly on formulation-stabilization of drug nanosuspensions, either disregarding or paying little attention to the impact of the process-equipment parameters, fundamental process understanding, process optimization-intensification, and process modeling. The review papers by Merisko-Liversidge *et al.* [42], Merisko-Liversidge and Liversidge [22], and Merisko-Liversidge and Liversidge [48] focused on formulating poorly water-soluble drugs as nanosuspensions, the applications of nanosuspension formulations, and the advantages of WSMM process for improving drug performance as well as patient compliance. Kesisoglou *et al.* [51] described the principles behind wet media milling, characterization of nanosuspensions as well as the current experience with *in vivo* utilization of such nanosuspensions. Shegokar and Müller [52] reviewed briefly the production technologies in industry and highlighted the great potential of nanoparticles for use in various application routes. The short review paper by Cooper [27] focused mainly on the applications of nanoparticles in different routes such as oral, injectable, inhalation, *etc*. The physical and chemical stability of drug nanoparticles, general stability issues, and common strategies to overcome stability issues were summarized by Wu *et al.* [53]. Wang *et al.* [54] discussed, in their review, unstable suspensions as well as methods and guidelines for selecting and optimizing stabilizers. Junyaprasert and Morakul [55] discussed the advantages of nanoparticles to improve *in vivo* performance, *i.e.*, pharmacokinetics, pharmacodynamics, safety and targeted delivery and described transformation of nanoparticles to final formulations and future trends of nanoparticles. Only Peltonen and Hirvonen [56] summarized various process parameters and their ranges investigated in wet media milling processes. While Peltonen and Hirvonen [56] and the references cited therein provide good understanding of the impact of stabilization and formulation, little fundamental understanding of the impact of process parameters has been provided in the absence of process models. Hence, it is fair to state that the aforementioned reviews are devoid of mechanistic, first-principle-based understanding of the wet media milling process for the production of drug nanosuspensions; process models, e.g., population balance models (PBMs) and microhydrodynamic models for better process understanding have not been covered.

The major objective of this review paper is to perform a review of pharmaceutical wet media milling literature in the last decade with a holistic focus on both the stabilization (formulation) and processing aspects with significant insight from emerging modeling studies. Firstly, formulation aspects with the commonly used stabilizers as well as novel stabilizers are covered and the stabilization mechanisms associated with these different classes of stabilizers are indicated. Secondly, complex and elusive effects of various process-equipment parameters are elucidated by microhydrodynamic process models, which enable not only the development of a fundamental understanding of the wet media milling process, but also provide guidance/insight for eventual process optimization, intensification, and scale-up. Finally, challenges and opportunities in the production of drug nanoparticles via wet media milling are discussed.

## 2. Formulation Aspects in the Preparation of Stable Drug Nanosuspensions

Drug suspensions produced by wet media milling should be physically stable during milling and storage for proper downstream processing and adequate shelf-life. Physical stability of the suspensions is broadly defined as the absence of a significant amount of aggregates in the suspensions and insignificant size increase/growth during the storage following wet media milling. In general, two major competing mechanisms operate during the size reduction of poorly water soluble drugs: particle breakage due to mechanical stresses and aggregation due to highly attractive inter-particle forces (van der Waals, hydrophobic forces, *etc*.) [57,58] (see Figure 2). In addition, Ostwald ripening may occur at a sufficiently high rate to cause particle growth [59]. Ostwald ripening is a process where the differences in solubility, as a function of the particle sizes, lead to a transport of material from small to larger particles with an accompanying size (growth) increase over time. Ostwald ripening may also be related to surface amorphization or mechano-chemical activation of the drug crystals due to the high stress provided by the beads [60]. The amorphous content might then dissolve immediately and recrystallize onto the drug crystals present.

**Figure 4:**
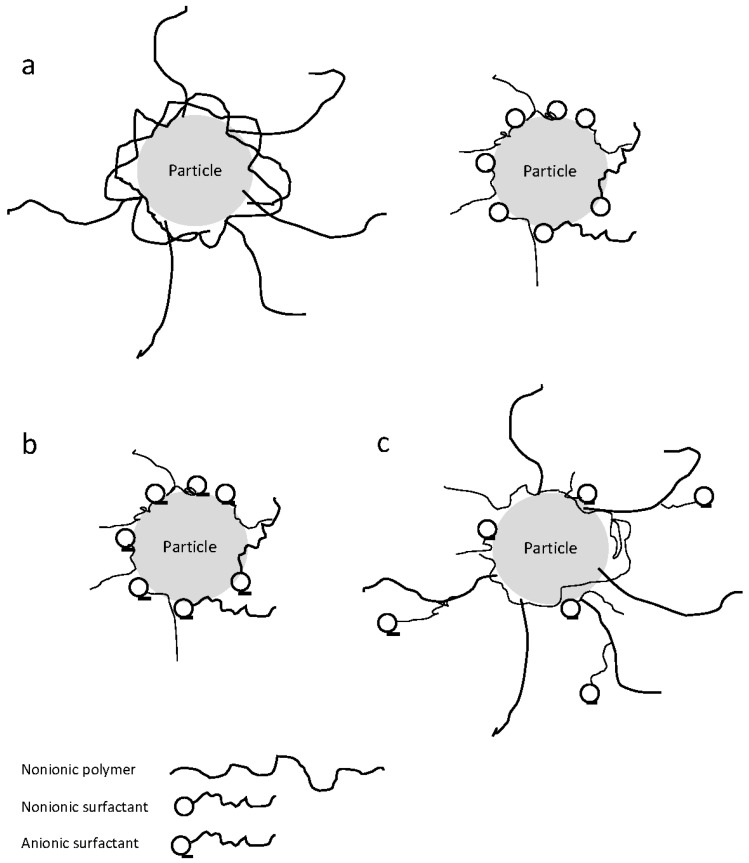
Schematic of physical stabilization mechanisms in drug nanosuspensions: (**a**) steric stabilization imparted by nonionic polymers or nonionic surfactants; (**b**) electrostatic stabilization imparted by anionic surfactants; and (**c**) electrosteric stabilization imparted by both nonionic polymers and anionic surfactants.

Selection of proper stabilizers and their optimum concentration plays a major role in formulating a stable drug nanosuspension. Inadequate concentration of stabilizer may not prevent drug nanoparticle aggregation, while its excess may promote Ostwald ripening. Electrostatic interactions, steric forces, entropic forces, and van der Waals forces among nanoparticles determine the overall physical stability of a drug nanosuspension [53]. A prerequisite for adequate stabilization is that the drug particles are wetted by the stabilizer solution and the stabilizer molecules (polymer or surfactant) have to adsorb onto the drug particle surfaces, e.g., by the anchor segments of the polymer and the hydrophilic or hydrophobic moiety of the surfactant (Figure 4).

Drug particles dispersed within a liquid continuous medium are stabilized by steric, electrostatic mechanisms, or by a combination of both (*i.e.*, electrosteric mechanism) via polymers and/or surfactants [31,48,56]. Steric stabilization is usually imparted by nonionic polymers and nonionic surfactants, e.g., cellulose derivatives, poloxamers (also considered as polymeric surfactants), polysorbates, and povidones, preventing particles from getting into the range of attractive van der Waals forces. Electrostatic stabilization is usually imparted by ionic surfactants, e.g., sodium dodecyl sulfate (SDS), dioctyl sulfosuccinate sodium salt (DOSS) and benzethonium chloride (BKC), providing mutual repulsion of similar charged particles. In electrosteric stabilization, nonionic polymers/surfactants and ionic surfactants act simultaneously.

A summary of recently published literature on drug nanoparticle stabilization during wet media milling is presented in Table 1. The data in Table 1 overall suggest that wet media milling has been used effectively to prepare nanosuspensions of a multitude of poorly water-soluble drugs, and various polymers and/or surfactants can be used for ensuring the adequate physical stability of the nanosuspensions. Interestingly, only three out of 43 studies reported in Table 1 achieved final drug particle sizes below 100 nm. In fact, only a few studies discussed the production of “true drug nanoparticles”, *i.e.*, particles with sizes ˂100 nm [6,61,62]. Hence, there is a huge gap in pharmaceutical nanotechnology literature regarding the preparation of true drug nanoparticles via wet media milling. Another finding is that a first-principle-based predictive method to select proper stabilizer(s) for a given drug is still missing; rather, the selection of stabilizer(s) appears to have been performed mostly empirically. Rather than attempting to develop first-principle-based predictive methods, recent studies have focused on developing more streamlined and material-sparing approaches to conserve time, effort, and materials. Juhnke *et al.* [63] developed a screening media mill equipped with up to 24 milling beakers which can perform up to 24 experiments in parallel to identify suitable drug stabilizers. Knieke *et al.* [64] developed a method called dynamic equilibrium curves, based on step-wise addition of stabilizers at the end of milling [65] and the concept of dynamic equilibrium [66], for investigating the physical stability of drug nanoparticle suspensions. Via this approach, optimal surfactant concentration was obtained from a single wet media milling experiment, which resulted in 75% time saving and 83% drug saving. Similarly, a drug-sparing approach was developed with the use of acoustic vibratory media milling with flexible choice of small processing vessels [67,68]. Finally, several studies measured stabilizer adsorption on the particles of various drugs and constructed adsorption isotherms, which were then used to guide a rational selection of the polymeric stabilizers in formulation development [30,64,69,70,71,72]. These studies showed that the polymer adsorption on drug surfaces increased with higher polymer concentration in the stabilizer solution; however, the slope of the adsorption isotherms decreased and a plateau (saturation) emerged, which can be described by a Langmuir adsorption isotherm. Practically, these findings suggest that beyond a certain level, the use of higher polymer concentration may not help to further stabilize drug nanosuspensions. Moreover, considering the negative effect of higher polymer concentration on breakage kinetics via viscous dampening [69], an optimal polymer concentration may be justified. On the other hand, the choice of polymer (stabilizer) concentration must also consider other factors such as the attributes and performance of final products (solid dosages or parenterals). Since this review mainly focuses on the production of stable drug nanosuspensions, the impact of stabilizers on downstream processing and final dosage attributes will not be covered here, and the readers are referred to other reviews with such focus, e.g., References [22,27,42,48,51,55,56].

### 2.1. Impact of the Physicochemical Properties of Drugs

Surface energy, solubility, molecular weight, type and number of functional groups, crystal structure, mechanical, and thermal properties of the drug crystals may all have an effect on the particle size distribution of the produced nanosuspensions in a wet media milling process. Few attempts have been made so far to understand the feasibility of nanosuspension formulation in terms of the mechanism of stabilization and various drug properties. As the literature does not provide any rational criteria for the selection of excipients and process conditions, formulation development was performed empirically [57,88,94,98]. To understand the impact of most critical drug properties on the stabilization of drug nanosuspensions, George and Ghosh [84] investigated the correlation between drug–stabilizer properties and critical quality attributes (CQAs) of the nanosuspension formulation. It was concluded that log*P* and fusion enthalpy have a direct correlation to the feasibility of formation of a stable nanosuspension. The most likely candidate for media milling is a drug substance with a high fusion enthalpy and hydrophobicity which can be stabilized either electrostatically or sterically. Also, the choice of an ideal stabilizer, *i.e.*, polymer/surfactant, was found to be influenced by the degree of hydrophobicity of the drug itself.

Choi *et al.* [103] investigated the stabilizing efficiency of polymers as a function of surface energies of the polymer and the drug. They concluded that not only the surface energy but also the specific interaction between the stabilizer and the drug appeared to play an important role in the stabilization ability of polymers. Lee *et al.* [98] formulated drug nanosuspensions containing polymers with varying chain length. Their study suggests that drugs with high molecular weights, low solubility, and high melting points, and surface energies similar to that of the stabilizer used could be successfully processed into nanosuspensions of unimodal particle size distribution. Verma *et al.* [71] demonstrated the use of atomic force microscopy (AFM) in visualizing the adsorption morphology of the drug substance to gain surface coverage and adhesion information, and used this methodology as a means of selecting suitable stabilizers in the production of a stable nanosuspension. Another study by Lee *et al.* [104] showed the role of specific interactions between the functional groups of stabilizers and drug particle surfaces.

In contrast to the aforementioned studies, a more comprehensive study [94], which used 13 stabilizers at three different concentrations to stabilize nine drug compounds, concluded that no correlation between physicochemical drug properties (molecular weight, melting point, log*P*, solubility and density) and stable nanosuspension formation exists. Similarly, a recent study [70] with five drugs found that there was no statistically significant correlation between the size reduction ratio for milling or the size growth ratio for storage stability and the physico-chemical drug properties (molecular weight, melting point, log*P*, solubility). Hence, elucidation of the impact of various drug properties is still elusive and warrants further investigation in the years to come.

### 2.2. Impact of Polymers as Stabilizers

Some water-soluble polymers adsorb on drug particle surfaces significantly during milling and reduce the extent of nanoparticle aggregation. Moreover, they reduce the interfacial tension between the hydrophobic drug particles and water [105,106]. Commonly used steric stabilizers include hydroxypropyl methyl cellulose (HPMC), hydroxypropyl cellulose (HPC), polyvinylpyrrolidone or povidone (PVP K-30), methyl cellulose (MC), hydroxyl ethyl cellulose (HEC), *etc*. A screening study conducted by Van Eerdenbrugh *et al.* [94] on 13 different stabilizers revealed that semi-synthetic polymers, e.g., HPMC, HPC, MC, HEC, carboxymethylcellulose sodium salt (NaCMC), and alginic acid sodium salt (NaAlg) showed a poor stabilizing performance among polymers investigated, while the linear synthetic polymers PVP K30 and PVP K90 exhibited better stabilizing potential when applied in higher concentrations. Obviously, the relatively low viscosity of PVP K30 based drug nanosuspensions allowed for the use of higher polymer concentrations as compared with the specific grades of cellulosic polymers used in that particular study. However, the above finding cannot be generalized because other grades of the semi-synthetic polymers could be used at much higher concentrations similar to PVP K30 grade, which could have enhanced the physical stability. In fact, a cursory look at the stabilizers used in Table 1 clearly shows that all of the above polymers and/or surfactants (see Section 2.3) can be successfully used to impart physical stability to drug suspensions. Also, for some drugs, some of the semi-synthetic polymers may impart better physical stability than PVP K30 in the presence of surfactants as co-stabilizers. Hence, the use of semi-synthetic polymers cannot and should not be ruled out from formulation development with drug nanosuspensions.

Besides the choice of the pharmaceutically acceptable polymer, formulators have to decide the polymer concentration/polymer:drug mass ratio that ensures a stable drug nanosuspension. A suitable working range of this ratio has been reported to be from 0.05:1 to 0.5:1 [51]. Lee *et al.* [98] compared HPMC, HPC, polyethylene glycol (PEG), and PVP at concentrations of approximately 17% relative to the concentration of drug. More recently, Van Eerdenbrugh *et al.* [94] indicated that higher PVP concentrations, *i.e.*, 25%–100% relative to the drug, produced more favorable milling results. However, the overuse of polymer may induce high viscosity in drug suspensions and thus cause viscous dampening during the milling, reducing the apparent breakage rate [64,69,80]. Hence, an optimal polymer concentration can be used to ensure physical stability of the drug nanosuspensions without causing excessively long milling times due to viscous dampening. Interestingly, in the absence of surfactants, much higher polymer (HPMC) concentration (polymer to drug ratio greater than 1:2) was needed to stabilize griseofulvin and fenofibrate nanosuspensions [65,107]. In fact, HPMC or HPC [69] was not able to completely disperse the primary griseofulvin nanoparticles that were produced during the milling. Other than the conventionally used polymers mentioned above, Yuminoki *et al.* [82] reported the application of Povacoat^®^, a hydrophilic polyvinylalcohol copolymer, which prevented the effective aggregation of various poorly water-soluble drug nanoparticles.

The physical stability can also be affected by the molecular weight of the polymer used [96]. An increase in molecular weight generates two counteracting effects: a decrease in the diffusion rate of polymer chains in a solution and an increase in the physical adsorption of the polymer. The effects on particle size reduction were more pronounced with polymers having lower molecular weights, and the effects of different molecular weights disappeared upon prolonged milling. The study concluded that the kinetic aspects of polymer molecular weight are important. Hence, Choi *et al.* [96] suggest that a polymer with a lower molecular weight is more suitable for efficient stabilization in wet media milling.

### 2.3. Impact of Surfactants as Stabilizers

Surfactants are compounds which lower the surface tension (or interfacial tension) and enable proper wetting of the drug particles with suspension liquid, usually water. In general, some of the polymers used in nanomilling can also reduce the interfacial tension and enhance wettability, but they do not allow wetting of surfaces as effectively as surfactants, e.g., SDS. Surfactants are classified according to their polar head group. The head of an ionic surfactant carries a net charge. If the charge is negative, the surfactant is more specifically called anionic, e.g., SDS, DOSS; if the charge is positive, it is called cationic, e.g., arginine hydrochloride (AH), BKC. If a surfactant contains no charge, it is called neutral or non-ionic, e.g., poloxamer 188/407 (also noted as pluronic F68/F127; it is a non-ionic block co-polymer), d-alpha tocopheryl polyethylene glycol 1000 succinate (Vit-E TPGS), and Polysorbate 20 and 80.

Bhakay *et al.* [58] reported that SDS is more effective than HPC in stabilizing the griseofulvin suspensions and all suspensions produced with SDS were relatively stable. SDS allows proper wetting of the hydrophobic griseofulvin particles and deaggregation of the aggregates. It also stabilizes the nanoparticles via an electrostatic mechanism as nanoparticles are formed during the milling. Bitterlich *et al.* [59] conducted a screening study for two poorly water-soluble drugs, fenofibrate and cinnarizine, to identify suitable formulations by employing commonly used polymers and surfactants. The addition of surfactants (DOSS or SDS) improved the stability significantly for all stabilizers investigated. For cinnarizine, no stable suspensions could be obtained at all without surfactants.

Poloxamer 338 and Vit-E TPGS were used by Baert *et al.* [91] to develop long-acting injectable formulations with rilpivirine (TMC278) nanoparticles for HIV treatment. Sufficiently stable, homogeneous, and resuspendable nanosuspensions could be prepared using both poloxamer and Vit-E TPGS. A similar observation was made by Van Eerdenbrug *et al.* [23,94] and Ghosh *et al.* [88] in that Vit-E TPGS was able to stabilize several drugs successfully. It was observed by Ghosh *et al.* [88] that Vit-E TPGS produced smaller particle sizes as compared to poloxamer (188 or 407) or SDS. However, Poloxamer 188 was reported to be the most versatile surfactant in terms of particle size reduction [98]. In terms of the impact of synthetic copolymers in stabilizing drug nanosuspensions, poloxamer 188 and Vit-E TPGS were reported to be more effective than semi-synthetic polymers and linear synthetic polymers [94].

It is noted that if surfactant concentration is used above the critical micelle concentration (CMC), drug solubility in the suspension could increase significantly, which could accelerate Ostwald ripening during storage [64,70]. Bitterlich *et al.* [59] found that the formulation with DOSS and cinnarizine resulted in a nanosuspension after milling. However, the storage stability was poor and cinnarizine particles grew fast even after a few hours of storage, as the high surfactant concentration promoted Ostwald ripening. Knieke *et al.* [64] had a similar observation for a formulation with wet-milled fenofibrate in the presence of HPMC–SDS.

### 2.4. Synergistic Stabilization via Combination of Polymers–Surfactants

The combination of HPC and SDS is known to have a synergistic effect in the stabilization of drug suspensions [28,45,58,69,70,92,108]. Polymers and surfactants not only allow proper stabilization of the nanoparticles in the suspensions, but they also do facilitate drug particle breakage [85]. Cerdeira *et al.* [57] attempted to identify the minimal use of polymer-surfactant for proper suspension stabilization and showed that excellent wetting of drug particles as well as electrostatic and steric stabilization by stabilizers is necessary to produce stable nanosuspensions via wet media milling. They found that a formulation with 0.0125% SDS and 3.125% HPC LF were required for the stabilization of miconazole nanosuspension. Other combinations of polymer and surfactants, besides HPC–SDS, were also studied such as HPC–Arginine hydrochloride (AH) [95], PVP–SDS [57,86], HPMC–SDS [57], HPMC–BKC [57], Poloxamer–SDS [83], and PVP–Polysorbate [90]. SDS was found efficient in combination with HPC, HPMC, PVP, and poloxamer 407 for various drugs such as prednisolone acetate, nifedipin, hydrocortisone acetate, itraconazole, azodicarbonamide, fenofibrate, griseofulvin, ibuprofen, and phenylbutazone [70,98]. But, the use of surfactant has to be optimized [57,70] because high concentration of SDS (>0.05%) was found to be detrimental for suspension stability. Besides the Ostwald ripening (see Section 2.3), this finding might be explained by the competitive displacement of adsorbed HPC by increasing SDS concentration [98,109,110]. Similar observations were made by Knieke *et al.* [64] for the milling of fenofibrate in the presence of HPMC–SDS and Bilgili *et al.* [70] for ibuprofen in the presence of HPC–SDS.

HPC adsorption was measured for different drugs with various HPC–SDS concentrations to gain a deeper understanding of steric stabilization imparted by HPC in the presence/absence of SDS [69,83], where synergistic stabilization with HPC–SDS combination was also explained by the higher HPC adsorption facilitated by SDS. In general, the adsorption of stabilizer onto drug particle surfaces is dependent on specific physicochemical interactions between the drug and stabilizer. For example, itraconazole nanoparticles exhibited approximately three times higher adsorption capacity for HPC (2200 µg/m^2^) than miconazole particles (750 µg/m^2^) [83]. Similarly, SDS adsorption onto the drug nanoparticles increased with increasing SDS concentration in the nanosuspensions [83]. When HPC and SDS are used in combination, they interact, forming aggregates or micelle-like SDS clusters bound to HPC [111]. These clusters can co-adsorb on drug surfaces [109,110], facilitating adsorption of HPC on drug particles [57], and enabling electrosteric stabilization [112].

### 2.5. Novel Stabilizers

#### 2.5.1. Colloidal Superdisintegrants

For some drugs, a soluble adsorbing polymer such as HPC, HPMC, PVP, *etc.*, alone may not be able to stabilize drug nanoparticles. As mentioned in Section 2.3 and Section 2.4, the use of surfactants either alone or in combination with the polymers can be helpful in addressing some limitations. There can be several issues associated with the use of surfactants such as physical instability of the drug suspensions [57], significant particle growth in suspensions via Ostwald ripening during storage especially if surfactant is used above CMC [88,113], and irritation to the pulmonary epithelium in inhalation applications [114,115,116]. Bhakay *et al.* [29] and Azad *et al.* [35,107] reported that anionic colloidal superdisintegrants, a class of cross-linked insoluble biopolymers, can be used as a novel class of stabilizers in the presence of an adsorbing neutral polymer. Such novel stabilizers allow for either minimizing or eliminating surfactants from the nanoparticle formulations. Croscarmellose sodium (CCS) and sodium starch glycolate (SSG) were used as anionic superdisintegrants in these studies. Azad *et al.* [117] showed that colloidal superdisintegrant particles can be produced by WSMM. The extensive particle breakage was attributed to the swelling-induced softening of these polymers in water. Griseofulvin and CCS/SSG particles were wet co-milled in the presence of soluble polymer HPC to produce surfactant-free stable suspensions [29,35]. SSG/CCS particles swell as they absorb water, which increase the nominal concentration of HPC in the aqueous solution. This increase in the nominal HPC concentration and increase in the volumetric solids loading due to larger volume of swollen SSG/CCS particles cause dramatic increase in the shear viscosity. The increased viscosity of the co-milled griseofulvin–HPC–CCS or SSG suspension reduces the mobility of the colloidal particles, thus imparting kinetic stability. Moreover, with an increase in the HPC solution concentration due to swelling, more HPC could adsorb on griseofulvin particle surfaces, thus leading to additional steric stabilization. Also, as observed in Azad *et al.* [107] and Bhakay *et al.* [29], the unmilled/shortly milled CCS particles could also contribute to the stability of the drug nanosuspensions by their swelling capability (leading to higher nominal polymer concentration) which helps to disperse drug aggregates under shearing. Azad *et al.* [107] thoroughly investigated the mechanisms by which colloidal superdisintegrants enhance the physical stability of the fenofibrate suspensions in the presence of HPMC, confirming the above mechanisms. Co-milled drug-superdisintegrant suspensions can be incorporated into nanocomposite microparticles (NCMPs) via fluidized bed coating/drying or spray drying [29,35] and into strip films [38] via wet film casting as surfactant-free drug delivery platform with the goal of enhancing the nanoparticle recovery and dissolution rate of poorly water-soluble drugs.

#### 2.5.2. Charged Nanoparticles

Charged nanoparticles (e.g., silica and polystyrene) combined with HPC were used by Juhnke and John [118] as a novel stabilizer to stabilize drug nanoparticles in a pH = 6 buffer. The addition of 0.1 wt % Latex SL (20 nm) resulted in a dramatically reduced apparent viscosity at low and high shear with only slight shear-thinning, *i.e.*, almost Newtonian fluid behavior. Remarkably, no particle aggregation of the colloidal drug suspension was detected after the addition of 1 wt % concentration Latex SL. The successful stabilization of drug nanosuspensions by charged nanoparticles in combination with an industrially relevant production technology opens up a new avenue in drug nanosuspension stabilization.

## 3. Processing: Impact of Process Parameters, Bead Material-Size, and Material Properties of Drug

Wet media milling is capable of producing stable nanosuspensions of a multitude of poorly water-soluble drugs; however, the process is time-consuming, costly, and energy-intensive [119]. While the pharmaceutical industry recognizes the aforementioned issues as well as other process related issues such as potential prdouct contamination due to bead wear, process-induced solid-state changes, and prolonged milling times needed to prepare drug nanosuspensions, a great majority of the published literature focuses on the stabilization of drug nanosuspensions [45,120]. Since the breakage kinetics determines the cycle time and production rate for a desired fineness, milling process design and optimization entails a good understanding of the breakage kinetics and its controlling process parameters. Process parameters such as stirrer/circumference/agitation speed, bead loading, and drug concentration can significantly affect the breakage rate and milling time required in a wet media milling process. Size and material of construction of the milling media (beads) also significantly affect the breakage kinetics and such parameters are usually treated as equipment design parameters because media are usually regarded to be part of the media milling equipment [119]. Another design related consideration is that both vertical and horizontal orientations of wet stirred media mills have been used for fine and ultrafine grinding of a wide range of materials [119]. There are also a multitude of vertical mill equipment designs, and the readers are referred to Kawatra [119] for different equipment design and operational details. On the other hand, the wet stirred media milling studies from 2008 to 2015 referenced in Table 2 used horizontal stirred mills, and the use of vertical stirred mills for nanomilling of poorly water-soluble drugs is not as common. Also, a head-to-head comparative assessment of the two mill orientations appears to be complicated, if not impossible. Hence, this review mainly focuses on the horizontal wet stirred media mills. A detailed discussion of the impact of each process parameter is given below.

### 3.1. Stirrer/Agitation Speed

**Figure 5:**
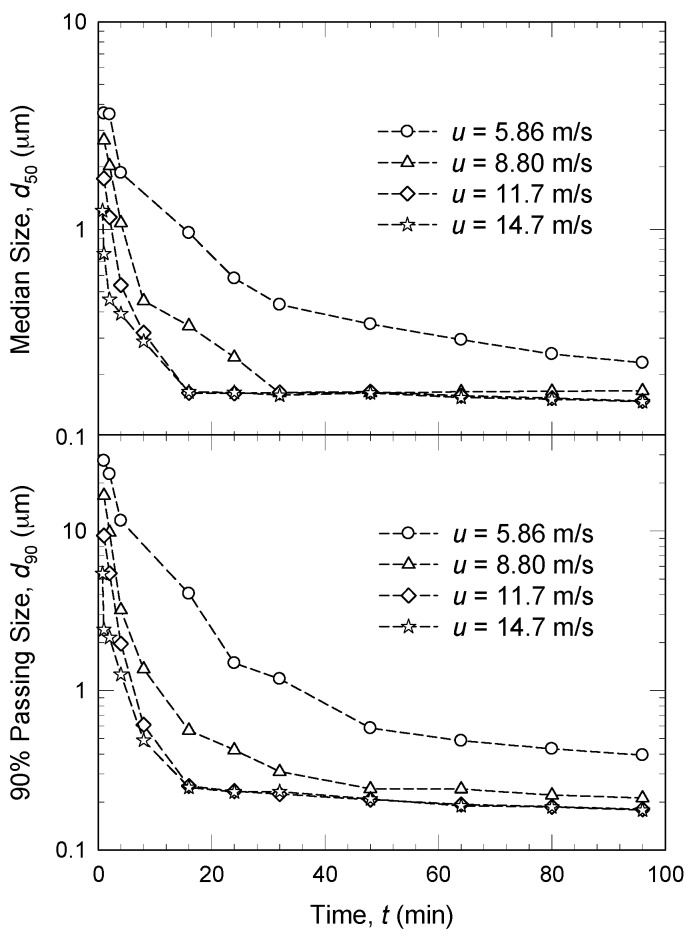
Effects of the stirrer speed (*u*) on the time-wise variation of the characteristic sizes of griseofulvin particles during wet stirred media milling (WSMM). Drug loading: 10% *w*/*w*, bead loading: 50 mL with a volumetric concentration of 0.388, bead size: 400 µm, and flow rate: 126 mL/min. Adapted from [45] with permission. Copyright Elsevier 2014.

The stirrer speed in WSMM or circumference speed in planetary ball mills plays an important role in determining the final particle size of the nanosuspensions [45,47]. Stirrer speeds of 2.65–14.7 m/s and circumference speeds of 150–6000 rpm have been reported [18,41,45]. The apparent breakage rate increases with an increase in the stirrer speed, as shown by the smaller median size (*d*_50_)/90% passing size (*d*_90_) at any given milling time during the WSMM process (Figure 5) [45]. Similar observation was reported by others [5,6,61,121]. For example, in Tanaka *et al.* [6], at a stirrer speed of 8 m/s, the probucol particles in the suspension were partially milled and a large fraction of the particles was still unbroken, while the fraction of unmilled particles significantly decreased upon an increase in the stirrer speed. At 12 m/s, all of the probucol particles were milled into the nanometer range (~139 nm). The effects of the stirrer speed at the beads scale were elucidated by Afolabi *et al.* [45] and Li *et al.* [61]. They calculated several microhydrodynamic parameters (see Section 4.3 for details) and concluded that the increase in the overall breakage rate upon an increase in the stirrer speed was explained by more frequent bead–bead collisions with greater stress intensity.

### 3.2. Bead Loading

**Figure 6:**
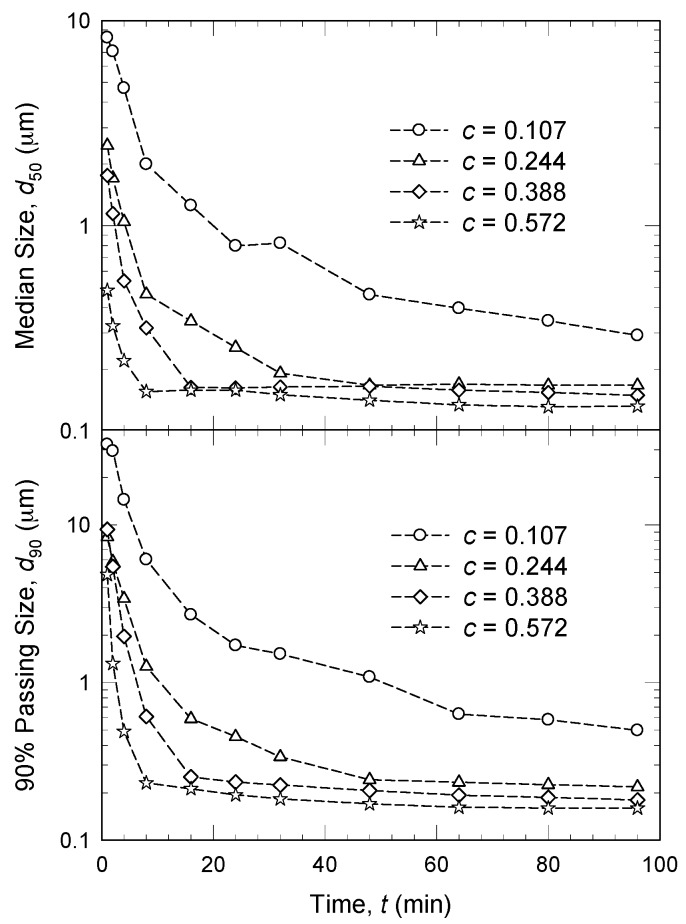
Effects of the bead concentration (*c*) on the time-wise variation of the characteristic sizes of griseofulvin particles during wet stirred media milling (WSMM). Drug loading: 10% *w*/*w*, bead size: 400 µm, flow rate: 126 mL/min, and stirrer speed: 11.7 m/s. Adapted from [45] with permission. Copyright Elsevier 2014.

Table 2 shows that the volumetric bead loading greatly varied in the literature from 17% to 94% of the milling chamber volume. The percentage is generally expressed in terms of apparent bead volume relative to the true milling chamber volume. Afolabi *et al.* [45] demonstrated that larger volume fraction of the beads resulted in faster breakage of griseofulvin particles (Figure 6). Other studies such as those by Patel *et al.* [90] and Li *et al.* [61] established the advantage of high bead loading in the attainment of fine drug nanoparticles. Patel *et al.* [90] suggests that the possible reason for the finer size is due to the smaller gap/void between the beads at higher bead loading, which prevents aggregation of the drug particles. Unlike Patel *et al.* [90], Afolabi *et al.* [45] and Li *et al.* [61] both prepared a physically stable drug nanosuspension that did not exhibit any aggregation; hence, attributing any bead loading effect to aggregation was ruled out. They explained the impact of bead loading using a microhydrodynamic model (see Section 4.3): an increase in the bead loading led to an increase in the specific energy consumption and the milling intensity factor, and consequently faster breakage. Upon an increase in the bead loading, the number of beads increases and the clearance between the beads decreases, leading to a dramatic increase in the bead–bead collisions and the average number of drug particle compressions per unit time. On the other hand, the fluctuating motion of the beads was less vigorous, which in turn led to smaller bead compression stresses [45]. The aforementioned analysis suggests that there exist two counteracting effects of the bead loading and such effects are complicated. Apparently, the former effect of the higher bead loading appears to dominate over the latter effect. Overall, at higher bead loading, faster breakage occurs as a result of dramatically increased drug particle compressions despite the reduction in the bead contact stresses.

### 3.3. Drug Concentration

**Figure 7:**
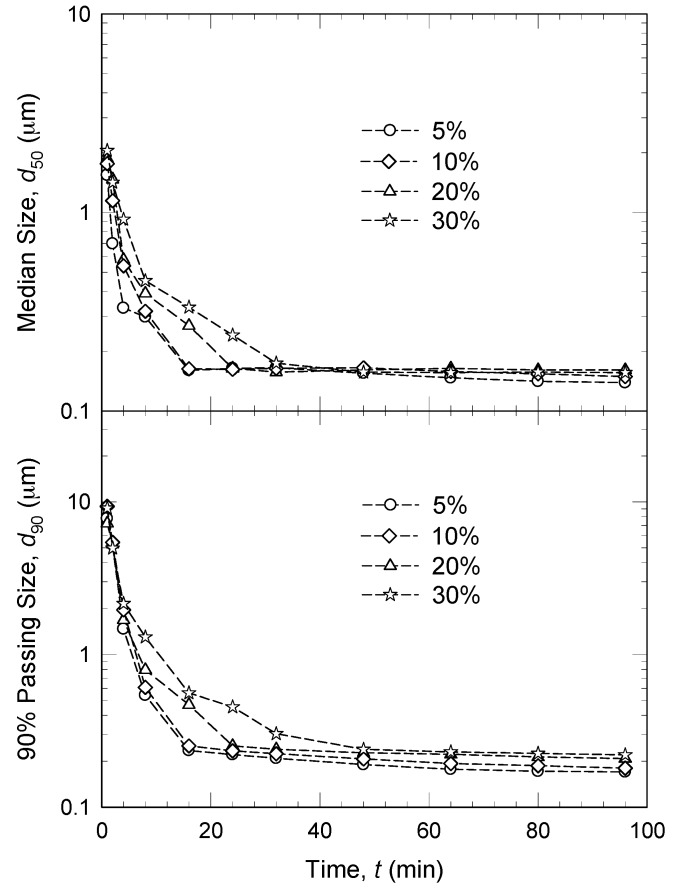
Effects of the drug loading on the time-wise variation of the characteristic sizes of griseofulvin particles during wet stirred media milling (WSMM). Bead loading: 50 mL with a volumetric concentration of 0.388, bead size: 400 µm, flow rate: 126 mL/min, and stirrer speed: 11.7 m/s. Adapted from [45] with permission. Copyright Elsevier 2014.

Afolabi *et al.* [45] found that an increase in griseofulvin concentration from 5% to 30% *w*/*v* led to a sharp decrease in the milling intensity factor and consequently slower breakage (see Figure 7). A similar observation was made by Ghosh *et al.* [47], where the drug concentration was investigated at two levels: 2% and 5% *w*/*v*. At higher drug concentration, a smaller fraction of nanoparticles was produced. While the breakage kinetics slowed down upon an increase in the drug concentration, more drug per batch was processed upon an increase in the drug loading. Interestingly, despite slower breakage, the time it took to mill per unit mass of the drug for a desired median size decreased significantly, signifying enhanced operational efficiency upon an increase in drug loading [45]. Considering the suspension preparation-unloading-cleaning times in-between multiple batches with e.g., 10% drug concentration along with the above breakage kinetics arguments, milling at a higher drug concentration (>10%) appears to be operationally more efficient, with appreciable reduction in the overall production time. The maximum drug loaded suspension reported in Table 2 is 44% *w*/*w*.

### 3.4. Size and Material of Construction of the Beads

As can be seen from Table 2, milling media (beads) made up of zirconia, alumina, or cross-linked polystyrene with various sizes have been used in wet media milling. Bitterlich *et al.* [59] reported that the milling performance was influenced by the choice of bead material and shape. Comparing zirconia and alumina beads of the same size, they concluded that zirconia beads transferred more energy per collision due to their higher density, therefore inducing faster breakage to drug particles during milling [73]. In addition, the milling process with spherical alumina beads was more efficient than the process with irregularly shaped alumina beads [59].

The reported nominal or median bead size found in the literature ranges from 15 to 1500 µm (see Table 2). Li *et al.* [61] investigated systematically the impact of bead size on breakage kinetics, final milled particle size, energy consumption, and bead wear. They found that the use of the smallest beads (50–100 µm) led to the fastest drug breakage, smallest drug particle size, significant energy savings, and lowest bead wear/product contamination. Similar observations were made by Cerdeira *et al.* [46] and Hennart *et al.* [121] in that smaller beads were more effective and achieved faster breakage. Via a microhydrodynamic model, Li *et al.* [61] showed that the use of smaller beads led to lower maximum contact pressure between the beads (unfavorable for breakage), but a dramatic increase in the average frequency of drug particle compressions (favorable). The overall impact of the bead size is expected to be dependent on which one of these two counteracting effects is more pronounced and how they relate to the mechanical properties of the specific drug. Apparently, in the aforementioned studies, the favorable effect of the smaller beads on the average frequency of drug particle compressions appeared to be the dominant factor, which was also reflected in the higher value of the milling intensity factor *F*. On the other hand, due to co-existence of these counteracting effects, it is not unreasonable to expect an optimum bead size for a particular set of wet media milling conditions, as found in some studies [68,88,125]. In fact, Li *et al.* [68] has recently demonstrated that not only did an optimal bead size exist, but also the optimal bead size decreased with an increase in the power density during the vibratory milling of griseofulvin. Obviously, without use of advanced models like the microhydrodynamic models (see Section 4.3 for details), one would not be able to elucidate these counteracting and elusive effects of the bead size.

### 3.5. Milling Time

Even at lab/small-scale, milling time for wet media milling processes can vary from 0.25 h to 1 day (see Table 2), which largely depends on the specific equipment-media-drug used, overall power density (specific energy consumption), and batch size. In general, wet stirred mills impart higher power density than planetary mills and rotating ball mills, and achieve the production of nanosuspensions faster. Regardless of milling equipment, a longer milling process usually allows for the production of smaller particles [5,47,97]. A longer milling is associated with more bead–bead collisions and drug particle compressions, which increases the extent of particle breakage and reduces the fraction of unmilled particles. By applying Box–Behnken design to the optimization of process parameters, Singare *et al.* [124] found that the minimum drug particle size was achieved at longer milling time and higher stirrer speed. On the other hand, particle sizes tend to approach a well-known milling limit or dynamic equilibrium provided that the milling is continued for a sufficiently long time [64,126]. In other words, prolonging the milling longer than what is needed for a desired drug particle size can cause unnecessary expenditure of energy/time; hence, overmilling should be avoided. Besides causing higher energy consumption, a prolonged milling could lead to unacceptable bead wear/contamination, and possible changes in the crystalline state of the drug, which will be discussed in Section 5.2. For lab-scale WSMM equipment, a minimum milling time of 0.5–1 h is generally required to obtain nanosuspensions with a unimodal particle size distribution having mean/median diameters below 200 nm [59,69,85].

### 3.6. Material Properties of Drugs

As mentioned in Section 2.1, physicochemical drug properties (molecular weight, melting point, log*P*, solubility and density) do not necessarily correlate well with the final milled particle size and physical stability of the milled suspensions. Hence, elucidation of the impact of various drug properties is still elusive and warrants further investigation in the years to come. A similar complexity arises when one attempts to correlate the mechanical properties of the drug to the milling performance. Unfortunately, a majority of the studies reported in Table 2 and in pharmaceutical nanotechnology literature, in general, did not attempt to correlate milling performance to mechanical properties of the drugs. Some qualitative trends connecting the milling performance to material properties has been indicated for the milling of other materials [119,127]. As a general guidance, softer materials and materials with higher brittleness index are easier to comminute [127]. Specifically for wet stirred media mills, the media (beads) material must be harder than the material to be ground in the mill; otherwise, the media will be subject to severe attrition/breakage [119]. Besides these qualitative trends, more elaborate and quantitative consideration of the material properties has been made in the context of various particle-scale mechanistic models. Such models explicitly incorporate various material properties to explain particle breakage (e.g., References [128,129,130,131]). Vogel and Peukert [131] derived two material parameters, *f*_Mat._ and *W*_m,min_, based on a dimensional analysis and fracture mechanical considerations to quantify the milling behavior of different inorganic materials. These parameters are respectively referred to as the material strength parameter and the threshold energy. The former denotes a material’s resistance against fracture under an external load and takes into account the particle’s relevant fracture and deformation mechanical parameters, while the latter is also a material property, *i.e.*, the energy that must be surpassed to initiate fracture either through single or multiple impacts. Meier *et al.* [132] showed that the breakage parameters are correlated to the ratio of hardness to fracture toughness of various sugars. This ratio was first suggested by Lawn and Marshall [133] to be indicative of a material's brittleness. In a follow-up study, Meier *et al.* [134] studied the impact breakage of nine different materials by impacting their particles on rigid targets at different velocities and found that the breakage function is both size and material dependent. In a study by Gahn and Mersmann [128], an attrition model was proposed, which seems to be sufficiently accurate for the modeling of attrition in crystallizers [129]. Ghadiri and Zhang [130] developed a mechanistic model of impact attrition of particulate solids having a semi-brittle failure mode to provide a basis to estimate the rate of attrition. A dimensionless attrition propensity parameter was derived, whereby the extent of breakage is related to the material properties, such as the particle density, a characteristic particle size, the hardness, and the fracture toughness as well as the impact conditions. While all of the aforementioned studies could be used to rank-order different materials in terms of their breakage propensity, they cannot predict the particle size distribution that results from a wet media milling process. Hence, there is a growing need to develop advanced multi-scale modeling approaches, which allow engineers to incorporate material properties of the drugs and media into existing milling process models for the prediction of the evolution of particle size distribution during the milling. Toward the development of such an advanced model, Afolabi *et al.* [45] utilized the microhydrodynamics of bead–bead collisions [49] and the Hertzian theory of elastic impact. Their microhydrodynamic model is capable of taking into account both the mechanical properties of the bead and material to be ground (see Section 4.3 for the model and the full set of references). On the other hand, to the best knowledge of the authors, no study has actually used the mechanical properties of the drugs in the context of this model because it is difficult to find reliable mechanical properties of the drug particles or to measure them [45,61]. Nonetheless, the microhydrodynamic model was instrumental in gaining fundamental insight into the impact of process parameters and the bead size.

## 4. Models for Enhanced Process Understanding

WSMM has proven to be a robust process for producing nanosuspensions of poorly water soluble drugs. As the process is expensive and energy-intensive, it is important to study the breakage kinetics, which determines the cycle time and production rate for a desired fineness. Singare *et al.* [124] and Singh *et al.* [5] used a statistical design of experiments with a response surface methodology [135] with the goal of optimizing the process parameters including stirrer speed and milling time. The statistically-based designs of experiments such as those in Singare *et al.* [124] and Singh *et al.* [5] allow for best use of experimental resources, good understanding of interaction effects, and selecting the optimal experimental conditions, but lack fundamental understanding of breakage kinetics and mechanisms. In general, milling dynamics, breakage kinetics, and scale-up effects have not been the major focus in pharmaceutical formulation studies [45,61,68]. Bhakay *et al.* [65] reported that most WSMM studies in pharmaceutical literature, e.g., Ain-Ai and Gupta [95] and Tanaka *et al.* [93], did not consider the breakage kinetics explicitly and focused mainly on the physical stability of the final milled suspensions. Other studies, e.g., Choi *et al.* [96], Deng *et al.* [97], and Lee *et al.* [98], reported limited analysis of the effects of milling time, with little to no quantification of the breakage kinetics and its relation to the microhydrodynamic phenomena occurring in the mill. Considering the importance of nanoparticle production for the enhancement of dissolution rate/bioavailability and the relatively expensive and energy-intensive nature of the WSMM, it is of utmost importance to study the breakage kinetics, which determines the milling time and production rates for a desired fineness of the drug product [45].

Mathematical modeling has been applied to the milling of various materials (see [136,137,138,139,140] and large body of literature cited therein); however, relatively scant modeling work has been reported for the nanomilling of drugs recently [45,61,69,141]. The energy consumed by a mill is an important quantity for media milling processes, but only a part of the energy is actually transmitted to the particles [68,142]. Kwade [142] developed theoretical considerations of interactions between the fracture energy spectra induced by bead motion to characterize the material properties and impact energy for stirred media mills. The impact energy spectra depend on the process conditions and can be determined by computing the collision energy via the discrete element method (DEM) [143] and simulating the hydrodynamics inside the milling chamber using computation fluid dynamics (CFD) codes based on Reynolds-averaged Navier-Stokes equations [144] or direct numerical simulation [145]. Another study by Gers *et al.* [146] estimated the capture probability of suspended particles by studying hydrodynamics in the gap between two milling beads. The bead motion was also examined by DEM simulations in the work conducted by Rosenkranz *et al.* [147]. In DEM simulations, the motion of each and every bead in a mill is tracked via the solution of the Newton’s law of motion in view of various contact mechanics laws. The DEM approach was originally introduced for geological investigations [148] and is nowadays applied in process engineering for the simulation of mills and other processes including various particulate systems [149,150,151,152]. However, a major challenge has to be overcome before DEM can realistically simulate a milling process and become predictive: the current computational power is insufficient to model the motion of all beads and material (drug) particles in a real mill [137]. In fact, the DEM simulations for milling processes have solely focused on the motion of the beads, disregarding the presence of material to be ground for the sake of computational necessity. Hence, in what follows, we will only focus on a few modeling techniques that can help to describe and explain the impact of process parameters on the breakage kinetics and drug particle size distribution.

### 4.1. Purely Descriptive Dynamic Models

Ample experimental data on the milling of various materials [153,154] suggest that the median particle size evolution exhibits first-order exponential decay in time *t*. Bilgili and Afolabi [45,69] fitted their experimental data via the following empirical model:

(1) $$d_{50}\left(t\right)=d_{lim}+\left[d_{50}\left(0\right)-d_{lim}\right]\exp\left(\frac{-t}{\tau_{p}}\right)$$

where *d*_50_(0) and *d*_lim_ denote the median size of the initial particle size distribution and the limiting particle size, respectively, while *τ*_p_ is a characteristic time constant of the milling process. Lower *τ*_p_ values correspond to faster breakage of the particles; hence, the impact of formulation-process parameters on the breakage kinetics can be analyzed via *τ*_p_. Fitting of the drug milling data was performed using SigmaPlot’s (Version 11) non-linear regression wizard, which uses the Marquardt–Levenberg algorithm to minimize the following sum of squared residuals (SSR):

(2) $$SSR=\overset{n}{\underset{i=1}{\sum }}{\left[log_{10}\left(d_{50i}^{exp}\right)-log_{10}\left(d_{50i}^{mod}\right)\right]}^{2}$$

where the superscripts “exp” and “mod” refer to experiment and model in Equation (1), respectively. The median size at different time points were indexed by *i* (*i* = 1, 2 ... *n*). To minimize the confounding effect of particle aggregation, the fitting analysis was performed on the drug suspensions with the polymer-surfactant combination, which were found to be more stable among the different formulations studied [58,69]. As the *d*_50_ values spanned a wide range, it became beneficial to take the logarithm of *d*_50_. The preliminary analysis indicated that large particles broke faster initially (usually within 1st min at the lab scale) than the particles produced after 1 min during WSMM. Therefore, Equation (1) with a single time constant *τ*_p_ was not able to fit the whole experimental data governed by two or potentially more characteristic time constants [155]. Hence, initial median particle size at 0th min was discarded, thus making the 1st min median size the initial size for better fitting capability, which is in line with prior studies [136,153,154].

### 4.2. Population Balance Models (PBMs)

Population balance models (PBMs) have been used as a tool for simulating, optimizing, and designing various particulate processes, including milling [156]. As a mathematical description of size reduction, it has been used extensively in literature [154,157,158,159,160,161]. PBMs have been used to simulate the milling of pigments [125,156,158,162] and minerals [163,164]. Given an initial (feed) particle size distribution (PSD), they have the ability not only to simulate the evolution of the PSD during a milling process, but they also have the ability to elucidate the breakage mechanisms such as fracture, cleavage, attrition [161,165,166] as well as the competing mechanisms like breakage–deaggregation–aggregation [140,162]. The application of PBM to pharmaceutical WSMM is limited and focuses mainly on the particle breakage [141]. While no PBM study has yet been reported for nanomilling of drugs that exhibit aggregation, this is not a serious obstacle for future studies in view of the available PBM literature on the wet media milling of non-pharmaceutical materials [140,162,164]. Despite having the aforementioned capabilities, PBMs are descriptive process-scale models, and without microdynamic information from particle scale, their predictive capability is rather limited. Moreover, PBMs do not explicitly account for the physics of any particle scale phenomena such as the bead–bead collisions that are critical to the understanding of a wet media milling process. Hence, this review paper mainly focuses on microhydrodynamic models.

### 4.3. Microhydrodynamic Models

In the context of WSMM, microhydrodynamics is the study of the fluctuating motion of the beads in sheared suspensions. Eskin *et al.* [49,167] developed a model to study the effects of stirrer speed and bead size on microhydrodynamics in a mixing tank filled with beads, which provided significant insight into the experimentally observed impact of these process parameters on the breakage rate. However, this model has not been applied to the wet media milling of drug suspensions with the goal of elucidating the impact of stabilizers or process parameters until recently. Knieke *et al.* [139] provided a semi-theoretical model based on kinematic equations that describe the relative motion of undeformed spheres (beads). However, that model does not allow calculation of the bead oscillation velocity and frequency, which fundamentally determine the breakage rate along with the drug material properties in WSMM. Bilgili and Afolabi [69] and Afolabi *et al.* [45] made the first attempt to further develop and apply the microhydrodynamic model developed earlier by Eskin *et al.* [167] to the WSMM of drug suspensions with the goal of understanding the beads’ oscillation and collisions and their impact on the breakage kinetics.

Based on the kinetic theory of granular flows and the fundamental granular energy balance [168], Eskin *et al.* [167] developed a model to calculate the mean velocity of the milling bead oscillations (fluctuations) in a well-mixed slurry. It is assumed that the power input by the mill stirrer is uniformly applied throughout the whole volume of the slurry and that it is equal to the total energy dissipation rate *ε*_tot_. The power input dissipates through fluctuating motions of the beads and liquid-beads viscous friction at the micro-scale; hence, the total energy dissipation rate (*ε*_tot_) is given by:

(3) $$P_{w}=\varepsilon_{tot}=\varepsilon_{visc}+\varepsilon_{coll}$$

where *P*_w_ is the power applied by the stirrer per unit volume, *ε*_visc_ is the energy dissipation rate due to both the liquid-beads viscous friction and lubrication, and *ε*_coll_ is the energy dissipation rate due to partially inelastic bead–bead collisions. With the consideration of energy dissipation due to friction between “liquid layers”, a new term (*ε*_ht_) should be added to the granular energy balance, as done in [169] for slurry flow in fractures. *ε*_ht_ is defined as the power spent on shearing equivalent fluid of the slurry (beads-drug suspension here) at the same shear rate but calculated or measured as if no particles (beads here) were present in the slurry. Then, following Eskin *et al.* [49] and Eskin and Miller [169], Equation (3) is modified by Afolabi *et al.* [45] as follows:

(4) $$P_{w}=\varepsilon_{tot}=\varepsilon_{visc}+\varepsilon_{coll}+\varepsilon_{ht}$$

(5) $$P_{w}=\frac{54\mu_{L}c\theta R_{diss}}{{d_{b}}^{2}}+\frac{12}{d_{b}\sqrt{\pi }}\left(1-k^{2}\right)\left[\frac{1-0.5c}{{\left(1-c\right)}^{3}}\right]c^{2}\rho_{b}\theta^{3/2}+\varepsilon_{ht}$$

where *µ*_L_ is the apparent shear viscosity of the equivalent liquid, *c* is the beads volumetric concentration, θ is the granular temperature defined as the bead-equivalent liquid relative mean-square velocity, *R*_diss_ is the dissipation or effective drag coefficient, *d*_b_ is the bead size, *k* is the restitution coefficient for the bead–bead collisions, and *ρ*_b_ is the density of the beads. The equivalent liquid properties *µ*_L_ and *ρ*_L_, the stirrer power per unit volume in the presence of the beads *P*_w_, and the energy dissipation rate for shearing the equivalent liquid *ε*_ht_ were measured. Along with the bead material properties, these measured values are incorporated into Equation (5), which can then be solved for the granular temperature θ using any non-linear equation solver, e.g., MATLAB’s fsolve function. The average bead oscillation velocity *u*_b_ and the frequency of single-bead oscillations *ν* are determined using the calculated θ and the following expressions:

(6) $$\mu_{b}=\sqrt{\frac{8\theta }{\pi }}\text{ and }v=\frac{24c}{d_{b}}\left[\frac{1-0.5c}{{\left(1-c\right)}^{3}}\right]\sqrt{\frac{\theta }{\pi }}$$

The energy dissipation rate resulting from the deformation of the drug particles per unit volume *Π* characterizes the milling intensity and is expressed as follows:

(7) $$\Pi =2.23\frac{c^{2}\left(2-c\right)}{{\left(1-c\right)}^{3}}\frac{1}{\pi^{5/2}\varepsilon \sigma_{y}}{\left(\frac{Y_{b}}{1-{\eta_{b}}^{2}}\right)}^{18/15}{\left(\frac{Y^{*}}{Y_{p}}\right)}^{\gamma }{\rho_{b}}^{4/5}\frac{R_{p}}{{R_{b}}^{2}}\theta^{13/10}$$

where *ε*, Y*, *Y*_p_, *η*_b_, *σ*_y_, *R*_p_, and *R*_b_ respectively denote volumetric drug concentration in the milled suspension, reduced elastic modulus of the bead–drug particle contact, elastic modulus of the drug particles, Poisson’s ratio of the beads, contact pressure in a drug particle captured when the fully plastic condition is obtained, radius of the drug particle, and radius of the bead.

To calculate *Π* using the expressions derived by Eskin *et al.* [167] (Equation (7)), one must either find the mechanical properties of the drug particles (*Y*_p_, *η*_p_, *σ*_y_) from the literature or measure them. Since it is difficult to find reliable mechanical properties of the drug particles or to measure them, Afolabi *et al.* [45] multiplicatively decomposed or factorized *Π* into a material-dependent factor *λ* and a process-dependent factor, *F*, which is referred to as the milling intensity factor: *Π* = *λF* with

(8) $$\lambda =2.23\frac{1}{\pi^{5/2}\sigma_{y}}{\left(\frac{Y_{b}}{1-{\eta_{b}}^{2}}\right)}^{18/15}{\left(\frac{Y^{*}}{Y_{p}}\right)}^{\gamma }{\rho_{b}}^{4/5}\frac{R_{p}}{{R_{b}}^{2}}$$

(9) $$F=\frac{c^{2}\left(2-c\right)}{{\left(1-c\right)}^{3}}\frac{1}{\varepsilon }\theta^{13/10}$$

Upon a change in each process parameter, a possible increase/decrease in *F* corresponds to a proportional increase/decrease in *Π* in some time- and space-averaged sense because well-mixedness and average power consumption during the milling are assumed, and *λ* may be regarded as a constant for given sizes of the specific drug–bead materials. One can then refer to either *F* or *Π* while reporting the impact of the process parameters. A similar factorization was performed by Li *et al.* [61] to explore the impact of bead size on *F*. Other important microdynamic parameters such as maximum contact pressure at the center of the contact circle formed between two colliding beads *σ*_b_^max^ and average frequency of drug particle compressions *a* can be calculated from respective mathematical equations (refer to [45]).

Bilgili and Afolabi [69], Afolabi *et al.* [45], Afolabi [120], and Li *et al.* [61] used the microhydrodynamic model to explain the impact of viscous dampening and various process-bead parameters during the WSMM of drug suspensions. The significant insights are as follows:

- Bilgili and Afolabi [69] found that there exits an optimal HPC concentration in WSMM of griseofulvin suspensions in the presence of HPC–SDS, which was explained by a combined microhydrodynamics-adsorption analysis. An increase in HPC concentration had two counteracting effects: reduction in *θ* at higher suspension viscosity (viscous dampening) and higher HPC adsorption on drug nanoparticles.
- Upon an increase in stirrer speed *u*, more mechanical energy was imparted and all microhydrodynamic parameters increased monotonically, *i.e.*, higher *u* led to higher *θ*, *ν*, *a*, *u*_b_, *σ*_b_^max^, and *F* [45,61,120]. In other words, higher *u* led to more frequent and energetic/forceful bead–bead collisions and more frequent drug particle compressions.
- An increase in volumetric bead concentration *c* led to two counteracting effects: *ν* and *a* increased, whereas *θ*, *u*_b_, and *σ*_b_^max^ decreased [45,61,120]. In other words, higher *c* led to more bead–bead collisions and drug particle compressions, but less energetic/forceful collisions/compressions. Overall positive impact, *i.e.*, faster breakage of the drug particles, was explained by an increase in the milling intensity factor *F*.
- An increase in drug loading led to a slight, almost linear decrease in all microhydrodynamic parameters, *i.e.*, *θ*, *ν*, *a*, *u*_b_, *σ*_b_^max^, except *F*, which exhibited a sharper decrease [45,61,120], thus explaining the reduced breakage rate.
- Similar to *c*, there were also two major counteracting effects of *d*_b_. A decrease in *d*_b_ led to lower *θ*, *u*_b_, *σ*_b_^max^ and higher *ν* and *a* [61], *i.e.*, more bead–bead collisions with less energy. The overall effect of *d*_b_ could not be explained by *F* alone; other microhydrodynamic parameters such as *ν* and *a* seem to explain the bead size impact better than *F*. While *F* can successfully explain the impact of all process parameters [45], it may be inadequate to explain the impact of bead size, which is usually regarded as an equipment parameter in media milling.

## 5. Challenges and Outlook

### 5.1. Preparation of Sub-100 nm Drug Particles

Nanoparticles of poorly water-soluble drugs, especially in the sub-100 nm range, have extremely large surface areas, exhibit higher saturation solubility owing to their increased curvature and dissolve faster [9,170]. In most pharmaceutical applications, there is a steady increase in demand for drug nanoparticles of lowest achievable size, as smaller particles offer improved permeation through the various biological barriers and also result in rapid onset of therapeutic action [52]. Moreover, drug nanoparticles with sizes less than 100 nm could render sterile filtration of aqueous drug nanosuspensions feasible and allow higher drug concentration for reduced injection volume in parenteral dosage forms [91,171].

Despite the use of various top-down and bottom-up approaches in the last few decades, only a small number of truly nanosized drugs having a median particle size below 100 nm are available in the literature [61,172]. Among the several bottom-up approaches, only the micro-emulsion method appears to be capable of producing sub-100 nm particles for multiple drugs [173,174]. However, this method suffers from various limitations such as low drug concentration, presence of organic solvents, and thermodynamically unstable particles. Contrary to bottom-up approaches, WSMM, a top-down approach, is considered more universal and promising for large-scale production of poorly water-soluble drug nanoparticles in suspension form because of its capability in achieving high drug concentration, organic solvent-free process, and continuous operation capability [42,85]. Li *et al.* [61] demonstrated, for the first time, the use of small beads in a model-guided intensified WSMM process for fast production of sub-100 nm particles of two BCS Class II drugs while achieving reduced energy consumption and keeping bead wear/contamination low.

### 5.2. Solid-State Changes

A concern regarding media milling of drugs is that it induces defects and potentially solid-state transformation, which in turn may affect material properties such as solubility. Several studies indicated that solid-state transformations took place during the dry milling of various drugs [175,176,177,178]. On the other hand, most studies on WSMM of drugs did not indicate such changes [35,61,68,107,116], except Kumar and Burgess [122]. Physical and chemical instabilities of naproxen (a poorly soluble drug) were investigated following low and high wet media milling intensity by Kumar and Burgess [122]. The naproxen-Polysorbate 80 formulations were stable regardless of the milling intensity, whereas naproxen–HPMC E15 wet-milled samples showed an infrared spectroscopy peak shift suggesting strong bond formation or molecular interaction, *i.e.*, amorphous phase. These results suggest that milling intensity and/or selection of stabilizers could be important to the preservation of the crystalline form of the drug in the wet-milled suspensions.

### 5.3. Contamination due to Media (Bead) Wear

In WSMM, wear occurs via impact and attrition due to collisions between beads–beads, beads–stirrer and beads–milling chamber, and contaminates the milled drug suspension. Owing to their high frequency, bead–bead collisions are the dominant type and result in the most wear. Although the contamination introduced by typical ceramic and polymeric milling beads is mostly not regulated by health authorities, they must be quantified in the lower ppm range, according to the current regulatory concepts and permitted daily exposures for oral, parenteral, pulmonary, and topical administration [123]. To reduce wear, equipment components stressed in the high-energy zone, such as beads, milling chamber and stirrer, are typically made from wear-resistant polymers and ceramics, e.g., highly crosslinked polystyrene and yttrium stabilized zirconia [31,48]. Nevertheless, contamination still occurs during milling and contaminants such as zirconium, yttrium, *etc.* are transferred to the drug products. Owing to the use of hard, wear-resistant materials along with judiciously selected milling process parameters, metal contamination due to bead wear can be kept under control at acceptable levels from low ppm to few hundreds of ppm level [61,123]. Regardless, contamination must be quantified for a specific mill, media, and drug to be milled, as part of a pharmaceutical development program. Practically, pieces of broken beads or defective beads can be removed by external sieving besides the mill’s internal separation system, e.g., a retention screen (refer to Figure 3). Due to repeated use of beads over a significant number of batches in manufacturing, the worn-out beads need replacement. In view of all of the above considerations, bead wear/contamination is currently highlighted as a major drawback for the production of nanoparticulate drug products by WSMM [52,56]. The contamination of the final product with heavy metals strongly increased with the increase of stirrer speed, bead size, and milling time [46,121]. The effect of the media milling parameters on the contamination level by different bead material has been investigated [179], where the contamination is related to the bead hardness. Juhnke *et al.* [123] reported that the wear is strongly dependent on the material and even the manufacturer of the milling beads. Li *et al.* [61] showed that the use of smaller beads led to lower zirconium contamination during WSMM because of the lower maximum contact pressure that results from bead–bead collisions. The WSMM process was intensified by making use of small beads (50–100 µm beads), which led to the fastest breakage of drug particles while keeping the specific energy consumption and media wear low [61].

### 5.4. Continuous Processing

A thorough search of pharmaceutical literature for wet media milling of poorly water-soluble drugs shows that a great majority of the experimental data was obtained with the batch/recirculation operation mode and scant information is available on the true (cascade) continuous mode of operation [85,180]. On the other hand, there is growing interest in moving all pharmaceutical unit operations in drug product manufacture from batch processes to continuous processes [181]. Monteiro *et al.* [85] suggest that the formulation, stabilization, and processing knowledge generated with batch/recirculation modes can be transferred to continuous mode without major difficulty. One can automate the multi-pass mode to allow passes between two holding tanks in a swing/pendulum fashion, which will save development time in predicting the number of mills required for the true (cascade) continuous milling operation. A cascade of 6–12 media mills in series can lead to production of drug nanosuspensions [85], while use of different bead sizes-processing conditions in different mills can reduce the number of mills required to a few.

### 5.5. Scale-up

A pharmaceutical development program may use ball mills, vibratory mills, planetary mills, *etc.* during the initial phase for identifying suitable nanosuspension formulations especially when available drug quantity is limited, whereas wet stirred media mills are commonly used at pilot-scale and manufacturing scale [47,59,63]. Wet stirred media mills are quite different in geometry, mode of operation, and power density than milling devices such as ball and planetary mills. Hence, moving from one type of equipment to another creates a challenge. For example, since the principle of agitation was completely different due to the presence of an impeller in the wet media mill as compared to the rotating jars in the planetary mill, a bridging study was needed to correlate the agitation rates between these two units [47]. Even though it is claimed that no significant difference was observed with regard to the particle size of nanoparticles between the two processes [47], the bridging was performed purely empirically. In general, it is strongly recommended that wet stirred media mills and vibratory media mills, which can impart high power density upon intensification similar to wet stirred mills [68], should be used at the initial phase of development, whereas wet stirred media mills can be used at pilot and manufacturing scales. In fact, Li *et al.* [68] demonstrated that an intensified vibratory milling process is suitable as a drug-sparing approach in early development and capable of preparing a similar nanosuspension to that prepared by wet stirred media milling, which is well-suited to pilot and manufacturing-scale production. In the scale-up of WSMM processes, similarity of the stirrer Reynolds number (*Re*), the stirrer Euler number (*Eu*) as well as the specific power consumption [119,120,182] can be used in scale-up. Afolabi *et al.* [45] suggests the potential use of various microhydrodynamic parameters such as the milling intensity factor *F* for process scale-up, but the validity of this approach is yet to be demonstrated.

### 5.6. Combined Methods

WSMM is a highly energy and time intensive process to produce drug nanoparticles. Additionally, product contamination due to media wear seems to be another constraint in the use of wet media milling [183]. While all of these issues can be overcome by developing enhanced process understanding of the wet media milling via various models such as the microhydrodynamic models (Section 4.3), alternative strategies have also been adopted in the milling literature. Patel *et al.* [184] combined WSMM and ultrasonication method to reduce fenofibrate particle size and improve stability of the prepared drug suspension. The approach of combining WSMM and ultrasonication method takes advantages of the individual methods; WSMM facilitates faster size reduction and ultrasonication in later stage results in reduced contamination of the product [184]. Similar combinative particle size reduction techniques were investigated in the pharmaceutical literature such as combining microprecipition and high pressure homogenization [185,186,187] and combining media milling and high pressure homogenization [188].

## 6. Summary

Preparation of drug nanoparticles via wet media milling (nanomilling) is a very versatile drug delivery platform and is suitable for oral, injectable, inhalable, and buccal applications. Wet media milling has become a well-established and proven formulation technology for bioavailability enhancement of poorly water-soluble drugs. It is a viable approach capable of resolving the issues associated with developing and commercializing poorly water soluble drugs and can be applied to practically any poorly water-soluble drug. This study has provided a comprehensive review of the pharmaceutical wet media milling studies mainly in the last decade, while covering both the formulation/stabilization aspects as well as processing aspects/issues in view of some advanced microhydrodynamic models. Unlike previous reviews, this review has provided a holistic perspective on the preparation of drug nanosuspensions considering both stabilization with conventional-novel stabilizers and processing. A review of the recently developed microhydrodynamic models has shed light and given significant insight into the impact of processing parameters, which is sorely lacking in previous reviews. Review of the formulation studies provides insight into the effective stabilization of drug nanoparticles to maintain their large surface area and ensure improved drug dissolution necessary for enhanced bioavailability. This review paper paves the way for streamlined process development scale-up based on microhydrodynamic models, while still indicating the need for development of first-principle-based predictive methods for stabilizer selection. Finally, various challenges and issues have been indicated with a view to shedding light on future opportunities.

## Figures and Tables

**Table 1:** List of publications on the stabilization of drug nanosuspensions produced via wet media milling between 2006 and 2015.

References (Year)	Drug	Drug Concentration (%) ^a^	Stabilizer(s) ^b^	Stabilizer Concentration (%) ^a^	Reported Smallest Median or Mean Particle Size after Milling (nm)
Bitterlich *et al.* (2015) [73]	Naproxen	5	Poloxamer-188, PVP-K 30, PVA-Mowiol 3-85, Mowiol 4-88, PVP-VA64, HPC-LF, Polysorbate-80, TPGS, SDS, HPMC	0.1–15	~150
Dong *et al.* (2015) [74]	SNX-2112	1	Poloxamer-188, Polysorbate-80	0.01–1	203
Kumar *et al.* (2015) [75]	Danazol	1	PVP-K 30, 40, PVA, HPMC-E3, E5, E15, Methocel-A15, SDS, TPGS, Poloxamer-188, 407, Dowfax-2A1, HPC	0.2	168
Afolabi *et al.* (2014) [45]	Griseofulvin	5–30 ^c^	HPC-SL	2.5	132
			SDS	0.5	
Bhakay *et al.* (2014) [76]	Griseofulvin, Azodicarbonamide	10 ^c^	HPC-SL, SDS	0–2.5	160
Bitterlich *et al.* (2014) [59]	Cinnarizine, Fenofibrate	10	DOSS	0.25	276
			SDS	0.1	
			Poloxamer-188, PVP-K30, PVP-VA64, PVA-Mowiol 3–85, Polysorbate-80, HPMC, Vit-E TPGS	2.5	
Komasaka *et al.* (2014) [77]	Cilostazol, Curucumin, Furosemide, Naproxen, Phenytoin, Nifedipine, Danazol, Spironolactone, Cinnarizine, Piroxicam, Indomethacin	10, 20	HPMC of different molecular weight, TC-5E, MC-400, Metolose, PVP-K17, Polysorbate-80, SDS, Cremophor-RH40, Poloxamer-188, Vit-E TPGS	0.5	~120
Leng *et al.* (2014) [78]	Paliperidone palmitate	15	Polysorbate-80	NM ^f^	492 (±8)
Mahesh *et al.* (2014) [79]	Glipizide	1.6	SDS, Poloxamer-188, 407, Polysorbate-80	2.5–7.5 ^d^	NM ^f^
			PVP-K30, HPMC	2.5–5 ^d^	
Shah *et al.* (2014) [80]	Glibenclamide	0.5	PVP-S630 D, Poloxamer-188, Polysorbate-80, HPMC, HPC, HEC, SDS	0.25	329
Sarnes *et al.* (2014) [81]	Itraconazole	15	Poloxamer-407	9–12	315 (±5)
Yuminoki *et al.* (2014) [82]	Griseofulvin, Hydrochlorothiazide, Tolbutamide, Acyclovir, Indomethacin, Diprydamole, Naproxen, Piroxicam, Phenytoin	1–20	HPC, PVP, POVA, PVA	1–10	120 (±2)
Bhakay *et al.* (2013) [58]	Griseofulvin, Phenylbutazone	10 ^c^	HPC-SL, SDS	0–2.5	145
			Mannitol	10	
Cerdeira *et al.* (2013) [83]	Miconazole, Itraconazole	5–20	SDS	0–0.2	136
			HPC-LF	1.25–5	
			HPMC-E15, Poloxamer-188, 407	5	
George and Ghosh (2013) [84]	Naproxen, compound A, B, C, D and E from Novartis	5	Vit-E TPGS, Poloxamer-407, SDS, DOSS	1	<500
			HPMC	2.5	
Knieke *et al.* (2013) [64]	Fenofibrate	2.5	HPMC-E3	5–50 ^e^	151
			SDS	5–20 ^e^	
Monteiro *et al.* (2013) [85]	Griseofulvin, Naproxen	10 ^c^	HPC-SL	2.5	138
			SDS	0.0825, 0.5	
Niwa and Danjo (2013) [86]	Phenytoin	8	PVP-K30	0.25–16	168
			SDS	0.1	
Ghosh *et al.* (2012) [47]	NVS-102	2, 5	HPMC	1	277
			Vit-E TPGS	0.5–5	
Tanaka *et al.* (2012) [6]	Probucol	1	Gelucire-44/14, Gelucire-50/13, Vit-E TPGS, Poloxamer-188, 338	1	77
Sievens-Figueroa *et al.* (2012) [36]	Naproxen, Fenofibrate, Griseofulvin	10^c^	HPMC-E15LV	2.5 ^c^	144
			SDS	0.075, 0.5^c^	
Ali *et al.* (2011) [87]	Hydrocortisone	2	PVP, Polysorbate-80	0.2	300
			HPMC	0.5	
Bhakay *et al.* (2011) [65]	Itraconazole, Fenofibrate, Griseofulvin, Ibuprofen, Azodicarbonamide, Sulfamethoxazole	2^c^	SA, SDS, HPMC, Polysorbate-80	0.1	740
			HPMC-E15 LV	0.2	
Cerdeira *et al.* (2011) [46]	Miconazole, Itraconazole, Etravirine	20	HPC-LF	5	129
			SDS	0–0.2	
Chin *et al.* (2011) [62]	Carbofuran	40.6, 44	Atlox-4913	4–7	29
			PVP-K30	1–3	
			Miglyol-812	1–3	
Ghosh *et al.* (2011) [88]	Compound NVS-102	5	Vit-E TPGS	3, 5	230.2
			SDS, HPMC, PVP-K30	1	
			Poloxamer-188, 407	2	
Liu *et al.* (2011) [89]	Indomethacin, Itraconazole	40	Polysorbate-80, PEG-6000, Poloxamer-188, 407	10–80 ^e^	345
Cerdeira *et al.* (2010) [57]	Miconazole	5–25	HPC-LF	1.25–6.25	140
			HPC-EF, PVP-30, Poloxamer-188, HPMC-E15	1.25, 2.5	
			SDS	0.0125, 0.05, 0.2	
			DOSS (SD)	0.1, 5	
			BKC	0.1	
Juhnke *et al.* (2010) [63]	Naproxen	2	HPC-LF	0.5	151
	Compounds A and B, from Novartis				
Patel *et al.* (2010) [90]	Famotidine	0.4	HPMC-K15M, PVP-K30, Polysorbate-80, Poloxamer-188, 407	0.4, 0.8	244.6
Baert *et al.* (2009) [91]	Rilpivirine (TMC278)	12.5	Poloxamer-338	3.125	200
			Vit-E TPGS	3.125	
Fakes *et al.* (2009) [92]	HIV-attachment inhibitor: BMS-488043	10	HPC-SL	1.25, 2.1	120
			SDS, DOSS	0.1	
Tanaka *et al.* (2009) [93]	Omeprazole, Albendazole, Danazol	1	Polysorbate-80, Poloxamer-188, 407	0.05–5	102
Van Eerdenbrugh *et al.* (2009) [94]	Loviride, Itraconazole, Cinnarizine, Griseofulvin, Indomethacin, Mebendazole, Naproxen, Phenylbutazone, Phenytoin	20	PVP-K30, K90, PVA-PEG (K-IR), Poloxamer-188, Vit-E TPGS, PVA, Polysorbate-80	10–100	>1000
			HPMC-E15, HEC, HPC, MC, NaCMC, NaAlg	1–10	
Ain-Ai and Gupta (2008) [95]	Naproxen	10, 30	HPC	1–4	417
			AH	0–1.2	
Choi *et al.* (2008) [96]	Itraconazole	8	HPC of different molecular weights	1.33	110
Deng *et al.* (2008) [97]	Compound A	15	Plasdone S-630	3.5, 4.1	82
			SD	0.25, 0.295	
Lee *et al.* (2008) [98]	Ibuprofen, Glimepiride, Digitoxin, Naproxen, Biphenyl dimethyl dicarboxylate, Paclitaxel, Lipoic acid, Predinisolone acetate, Nifedipin, Hydrocortihydrocortisone acetate, Itraconazole	8	HPC, PVP, PEG , Poloxamer-188, 407	1.33	119 (±37)
			SDS, Benzethonium chloride	1	
Van Eerdenbrugh *et al.* (2008) [23]	Loviride, Itraconazole, Cinnarizine, Griseofulvin, Indomethacin, Mebendazole, Naproxen, Phenylbutazone, Phenytoin	20^c^	Vit-E TPGS	25 ^e^	156
Dai *et al.* (2007) [99]	Poorly water soluble compound/carrageenan complex	5	Poloxamer-407	0.75	300
			Tyloxapol, HPMC-2910, HPC-SL	1.5, 2	
			PVP-K30	0.75, 2	
			Plasdone-S630	1.31, 2	
			DOSS	0.15	
Sepassi *et al.*(2007) [100]	Nabumetone, Halofantrine	20	HPMC-E3LV, E4M, PVP-K12, K30, K90	0.63–6.25	650
Van Eerdenbrugh *et al.*(2007) [101]	Loviride	20	Polysorbate-80, Poloxamer-188	50 ^e^	264 (±14)
Jinno *et al.* (2006) [102]	Cilostazol	0.25	HPC	16.5	220
			DOSS	0.8	

^a^ With respect to suspension, *w*/*v* or *w*/*w*; ^b^ Names of the stabilizers are abbreviated: AH: Arginine hydrochloride; Atlox 4913: Poly(methyl methacrylate) poly(ethylene glycol) graft copolymer; BKC: Benzalkonium chloride; DOSS (SD): Dioctyl sulfosuccinate sodium salt (sodium docusate); HEC: Hydroxyethylcellulose; HPC: Hydroxypropyl cellulose; HPMC: Hydroxypropylmethyl cellulose; MC: Methylcellulose; Miglyol 812: a 60/40 (*w*/*w*) mixture of C8 and C10 triglycerides; NaAlg: Alginic acid sodium salt; NaCMC: Carboxymethylcellulose sodium salt; PEG: Polyethylene glycol; Plasdone S-630: Copoviodone, Vinyl pyrrolidone/vinyl acetate copolymer; PVA: Polyvinyl alcohol; PVA-PEG: Polyvinyl alcohol polyethylene glycol graft copolymer; PVP: Polyvinylpyrrolidone; SDS: Sodium dodecyl sulfate; SA: Sodium alginate; Vit-E TPGS: d-alpha tocopheryl polyethylene glycol 1000 succinate; POVA: PVA copolymer with grafted poly acrylic acid and poly methyl methacrylate (PMMA) groups; ^c^ with respect to deionized water; ^d^ stabilizer:drug; ^e^ with respect to drug weight; ^f^ not mentioned.

**Table 2:** Summary of the process-equipment parameters investigated in wet media milling literature from 2008 to 2015.

References (Year)	Mill Type	Stirrer/Circumference Speed (rpm)	Suspension Flow Rate (mL/min)	Milling Time (h)	Bead Type ^a^	Nominal or Median Bead Size (µm)	Bead Boading (%) ^b^	Drug Concentration (%) ^c^
Bitterlich *et al.* (2015) [73]	Planetary ball mill	400	NM ^f^	4	Al_2_O_3_	100	50	5
					Al_2_O_3_	300		
					ZrO_2_	100		
					ZrO_2_	200		
					ZrO_2_	300		
					ZrO_2_	500		
Li *et al.* (2015) [68]	Vibratory media mill	40%–90% ^d^	NM ^f^	1.6	ZrO_2_	50–1500	30–70	10
Li *et al.* (2015) [61]	Wet stirred media mill	11.7–14.7 ^e^	126–343	2–6	ZrO_2_	50–800	62.5–93.75	10
Afolabi *et al.* (2014) [45]	Wet stirred media mill	5.86–14.7 ^e^	126	1.6	ZrO_2_	430	17.5–93.75	5–30
Kumar and Burgess (2014) [122]	Wet stirred media mill	2000–3400	NM ^f^	1–4	ZrO_2_	NM ^f^	NM ^f^	1
Shah *et al.*(2014) [80]	Wet media mill	400–1100	NM ^f^	3–11	ZrO_2_	100–1000	50 ^f^	0.5
Bitterlich *et al.*(2014) [59]	Planetary ball mill	400	NM ^f^	4	ZrO_2_	325	50	10 ^g^
	Wet stirred media mill	9 ^e^	NM ^f^	6–24	Al_2_O_3_ (irregular)	185–320	70 ^h^	
					Al_2_O_3_ (spherical)	311		
					ZrO_2_	185–475		
Monteiro *et al.* (2013) [85]	Wet stirred media mill	13.2 ^e^	55–110	~1	ZrO_2_	430	62.5	10
Ghosh *et al.*(2012) [47]	Planetary mill	150–400	NM ^f^	4	ZrO_2_	100–500	NM ^f^	2–5
	Wet stirred media mill	2500	NM ^f^	1–4	ZrO_2_	100–500	NM ^f^	
Juhnke *et al.*(2012) [123]	Wet stirred media mill	6–12 ^e^	NM ^f^	NM ^f^	ZrO_2_	100–500	80	10 ^g^
Tanaka *et al.*(2012) [6]	Wet stirred media mill	8–12 ^e^	NM ^f^	NM ^f^	ZrO_2_	15–50	500 ^i^	1
Bhakay *et al.* (2011) [65]	Wet stirred media mill	2.65 ^e^	NM ^f^	0.5–1.3	Crosslinked polystyrene	200–350	50	2
	Attritor mode	2.65–4.97 ^e^	NM ^f^	1.3	Zirconia rings	NM ^f^		
Cerdeira *et al.*(2011) [46]	High energy media mill	2400–3600	97–183 ^j^	0.25–1	ZrO_2_	400–800	81–85	20 ^g^
Chin *et al.*(2011) [62]	High energy intensive ball mill	3000	NM ^f^	2	ZrO_2_	100–800	NM ^f^	40.6–44 ^g^
Singh *et al.*(2011) [5]	Wet stirred media mill	2500–3400	100	3–6.5	ZrO_2_	200	NM ^f^	4
Hennart *et al.*(2010) [121]	Wet stirred media mill	2000–6000	NM ^f^	3	ZrO_2_	300–800	80	NM ^f^
Juhnke *et al.* (2010) [63]	Planetary mill	400	NM ^f^	0.25–2	ZrO_2_	200	60	2 ^g^
	Wet stirred media mill	10 ^e^	NM ^f^	8	Crosslinked polystyrene	360–500		
		6 ^e^		8	ZrO_2_	100		
Singare *et al.*(2010) [124]	Wet stirred media mill	2500–3400	100	3–6	ZrO_2_	200	NM ^f^	6.4
Deng *et al.*(2008) [97]	NanoMill-01 Systems milling apparatus	1800–4400	NM ^f^	0.67–1	Cross-linked polystyrene	500	NM ^f^	15 ^g^

^a^ ZrO_2_: yttrium stabilized zirconium dioxide bead, Al_2_O_3_: aluminum oxide bead; ^b^ filling volume fraction of bead bulk volume relative to the volume of the milling chamber, *v*/*v*; ^c^ with respect to deionized water; ^d^ intensity; ^e^ in m/s; ^f^ not mentioned or not applicable for the specific mill/mode of operation; ^g^ with respect to suspension, *w*/*w*; ^h^ weight of grinding media filling ratio; ^i^ in g; ^j^ in g/mL.

## Symbols Used

*a* average frequency of drug particle compressions, Hz; *c* volumetric concentration of the beads, –; *d* particle diameter (size), m; *d*_lim_ limiting particle size of the drug particles, m; *F* milling intensity factor, m^2.6^/s^2.6^; *k* restitution coefficient for bead–bead collisions, –; *P*_w_ power applied by the mill stirrer per unit volume, W/m^3^; *R* radius, m; *R*_diss_ dissipation (effective drag) coefficient of the bead, –;SSR sum-of-squared residuals; *t* milling time, s; *u*_b_ average bead oscillation velocity, m/s; *Y* Young modulus, Pa; *Y** reduced elastic modulus for the bead–drug contact, Pa.

## Greek Letters

*ε* volumetric fraction of drug particles in the drug suspension, –; *ε*_coll_ energy dissipation rate due to partially inelastic bead–bead collisions, W/m^3^; *ε*_ht_ power spent on shear of equivalent liquid of the slurry at the same shear rate but calculated (measured) as if no beads were present in the flow, W/m^3^; *ε*_tot_ total energy dissipation rate, W/m^3^; *ε*_visc_ energy dissipation rate due to both the liquid-beads viscous friction and lubrication, W/m^3^; *γ* a coefficient for bead–drug particle contact, –;*η* Poisson’s ratio, –;*λ* material-dependent factor, kg/m^3.6^·s^0.4^; *µ*_L_ apparent shear viscosity of the equivalent fluid, Pa·s; *ν* frequency of single-bead oscillations, Hz; *Π* energy dissipation rate attributed to the deformation of drug particles per unit volume, W/m^3^; *θ* granular temperature, m^2^/s^2^; *ρ* density, kg/m^3^; *σ*_b_^max^ maximum bead contact pressure at the center of the contact circle, Pa; *σ*_y_ contact pressure in a drug particle when the fully plastic condition is obtained, Pa; *τ*_p_ characteristic time constant of the process, s.

## Indices

b beads; p drug particle; 50 50% passing size (median size) of the cumulative distribution, m; L equivalent liquid (milled drug suspension).
